# CuZn and ZnO Nanoflowers as Nano-Fungicides against *Botrytis cinerea* and *Sclerotinia sclerotiorum*: Phytoprotection, Translocation, and Impact after Foliar Application

**DOI:** 10.3390/ma14247600

**Published:** 2021-12-10

**Authors:** Panagiota Tryfon, Nathalie N. Kamou, Stefanos Mourdikoudis, Katerina Karamanoli, Urania Menkissoglu-Spiroudi, Catherine Dendrinou-Samara

**Affiliations:** 1 Laboratory of Inorganic Chemistry, Department of Chemistry, Aristotle University of Thessaloniki, 54124 Thessaloniki, Greece; tryfon.giota@gmail.com; 2 Pesticide Science Laboratory, Faculty of Agriculture Forestry and Natural Environment, School of Agriculture, Aristotle University of Thessaloniki, 54124 Thessaloniki, Greece; nnkamou@gmail.com; 3 Biophysics Group, Department of Physics and Astronomy, University College London, London WC1E 6BT, UK; s.mourdikoudis@ucl.ac.uk; 4 UCL Healthcare Biomagnetics and Nanomaterials Laboratories, 21 Albemarle Street, London W1S 4BS, UK; 5 Laboratory of Agricultural Chemistry, Faculty of Agriculture, School of Agriculture, Forestry and Natural Environment, Aristotle University of Thessaloniki, 54124 Thessaloniki, Greece; katkar@auth.gr

**Keywords:** bimetallic CuZn nanoflowers, ZnO nanoflowers, nano-pesticides, phytopathogenic fungi, plant protection

## Abstract

Inorganic nanoparticles (INPs) have dynamically emerged in plant protection. The uptake of INPs by plants mostly depends on the size, chemical composition, morphology, and the type of coating on their surface. Herein, hybrid ensembles of glycol-coated bimetallic CuZn and ZnO nanoparticles (NPs) have been solvothermally synthesized in the presence of DEG and PEG, physicochemically characterized, and tested as nano-fungicides. Particularly, nanoflowers (NFs) of CuZn@DEG and ZnO@PEG have been isolated with crystallite sizes 40 and 15 nm, respectively. Organic coating DEG and PEG (23% and 63%, respectively) was found to protect the NFs formation effectively. The CuZn@DEG and ZnO@PEG NFs revealed a growth inhibition of phytopathogenic fungi *Botrytis cinerea* and *Sclerotinia sclerotiorum* in a dose-dependent manner with CuZn@DEG NFs being more efficient against both fungi with EC_50_ values of 418 and 311 μg/mL respectively. Lettuce (*Lactuca sativa)* plants inoculated with *S. sclerotiorum* were treated with the NFs, and their antifungal effect was evaluated based on a disease index. Plants sprayed with ZnO@PEG NFs showed a relatively higher net photosynthetic (4.70 μmol CO_2_ m^−2^s^−1^) and quantum yield rate (0.72) than with CuZn@DEG NFs (3.00 μmol CO_2_ m^−2^s^−1^ and 0.68). Furthermore, the penetration of Alizarin Red S-labeled NFs in plants was investigated. The translocation from leaves to roots through the stem was evident, while ZnO@PEG NFs were mainly trapped on the leaves. In all cases, no phytotoxicity was observed in the lettuce plants after treatment with the NFs.

## 1. Introduction

Approximately 70% of crop loss has been reported due to pathogenic fungal disease [1]. *Botrytis cinerea* and *Sclerotinia sclerotiorum* are plant pathogenic fungi that cause a significant decrease in crop production with serious economic losses [1,2]. *B. cinerea* has a broad host range, causing severe damage, in different stages of crop cultivation and commercialization. Although biocontrol means are currently used effectively, control methods of *Botrytis* are mainly based on chemical fungicides, which have been attributed to the rise of chemical resistance or unwanted effects on the surrounding agro-ecosystem [2,3]. *S. sclerotiorum* causes lettuce drop, an important disease, whose symptoms include damping-off, root rots, and wilting, and result in severe crop losses. There are a few lettuce varieties resistant in *Sclerotinia*, and control is reliant on ‘off-label’ use of fungicides, which target sclerotia [4]. Recently, plant protection by nanomaterials gives rise to the ability of site-specific use of pesticides in an eco-friendly approach. Nanopesticides are reported to be more potent when compared with analogs, facilitating a gain of maximum crop yield, providing targeted and controlled release action, and consequently minimizing the use of agrochemicals and reducing the final costs of crop production [5,6].

Inorganic nanoparticles (INPs) have dynamically emerged in plant protection, and various compositions have been reported as potential candidates for controlling a range of agricultural pests, such as fungi, bacteria [7,8,9], and nematodes [10,11]. INPs provide an alternative to conventional agrochemicals due to their greater solubility, mobility, and durability. Particularly, INPs with antifungal activity are effective at low doses, while the slow release of less toxic chemicals reduces the development of resistance and offers greater protection from quick degradation [12]. Additionally, when bioessential metals are incorporated into these nanoscale products, they may act synergetically as fertilizers, improving the plant growth and enhancing the plant tolerance of various biotic and abiotic stresses [12,13].

Among different compositions of INPs, Cu-based and zinc oxide nanoparticles (ZnO NPs) have been suggested as antifungals [14,15,16]. These metallic NPs are cost-effective and at low concentrations are considered eco-friendly and non-toxic, while they have bioactive properties and are also involved in plant growth, significantly enhancing the availability and displacement of micronutrients within plants [1]. Copper is regarded among the relatively safe agrochemicals, hence its use in organic farming. Cu-based formulations have been used as pesticides (fungicides and bactericides) for centuries, and they are sprayed in the form of copper sulfate, copper oxychloride, and copper carbonate. Copper is listed in USEPA(2008) as a pesticide and is extensively used in various agricultural practices. The annual application of copper in crop protection strategies is calculated at millions of tons. Moreover, ZnO constitutes an approved food additive by the Food and Drug Administration (FDA) [17], while biocompatibility studies of ZnO NPs revealed no significant toxicity either in cell lines or in skin infection [18]. Recently, Ali et al. reviewed the biological and environmental properties of ZnO NPs and reported on the extensive use of ZnO NPs in the medicinal industry, as a biosafe, biocompatible, and non-toxic nanomaterial [19]. Rationalization is still very difficult for a plethora of pathogens because of the variability of important factors such as *in vitro* and *in planta* bioassays, nanosizes, shapes, and morphologies of INPs. Thus, various structures of ZnO NPs, as spheroidal and needle-shaped, were shown to possess antifungal properties against postharvest pathogenic fungi including *B. cinerea* and *A. niger* [20], while other studies against *P. expansum* [21], *A. alternate*, and *F. oxysporum* [22] turned out to be very promising. Furthermore, photoactivated ZnO NPs (200 nm) inhibited the growth of *B. cinerea* by 58% at a concentration of 5 × 10^−3^ M 24 h after treatment [23]. Several antifungal studies incorporate Cu-based NPs where composition effects in addition with nanoeffects seem to be essential. For example, Cu-based NPs mainly with a spherical structure have been reported to display fungal inhibition toward *P. infestans* [7] and *B. fabae* [24] with Cu_2_O NPs exhibiting higher efficacy. Even though the different morphologies of INPs and the combination of metals can influence the antifungal activity, to date, a limited number of studies have evaluated the antifungal efficacy of nanostructures with more complicated structures and compositions such as bimetallic ones. Among different nanostructures, the flower-shaped structure, called nanoflowers (NFs), is composed of several particles forming flowers. Flower-shaped structures provide a higher number of adsorption sites than spherical nanoparticles and demonstrate better charge transfer and carrier immobility. The efficiency of their surface reaction is increased in the 3D structure, which plays a major role in the determination of surface enhancement [25]. Antonoglou et al. reported that nanoclusters of coated CuZn NFs caused fungal inhibition against *S. cerevisiae* with a negligible effect on photosystem II of tomato leaf [8].

In the present study, a modified polyol process under solvothermal conditions has been used for the synthesis of coated bimetallic (CuZn@DEG) and metal oxide (ZnO@PEG) NFs, which were further tested as nanofungicide agents *in vitro* and *in planta*. Agrochemical formulations of INPs in order to adapt as phytoprotection agents require the use of a surrounding surface organic coating (shell). Such a coating is not only essential for the preparation and maintenance of long-term physical stability of INPs, but it is also important for enhancing the biological performance of the agrochemical, increasing the foliar uptake of herbicides, growth promoters, and defoliants [26]. Generally, coating layers protects INPs from dissolution and oxidation, which can be commonly occurring processes for the INPs in aqueous medium. The chemical nature and the amount of the coating can offer new properties as hybrid structures are formed that improve the biocompatibility of the INPs, which is critical for biological exploitation [27]. In that vein, the glycol derivatives (polyols), diethylene glycol (DEG) and polyethylene glycol (PEG), respectively, have been used in a triple role, as solvent, stabilizing agent, and reducing agent in the synthesis, and they allow us to prepare coated, hydrophilic, and highly crystalline particles of NFs morphology. The physicochemical characteristics of the resulted hybrid ensembles, which are very important for the biological evaluation, were identified thoroughly by X-ray diffraction (XRD), thermogravimetric analysis (TGA), Fourier transform infrared spectroscopy (FT-IR), transmission electron microscopy (TEM), dynamic light scattering (DLS), *ζ*-potential, and ionic release analysis (ICP). The antifungal activity against two notorious phytopathogenic fungi *B. cinerea* and *S. sclerotiorum* has been investigated *in vitro*. *In planta* experiments included the pathosystem of lettuce (*Lactuca sativa* L.) and *S. sclerotiorum*, since lettuce is one of the most widely consumed vegetables. The synthesized NFs were applied on lettuce plants inoculated and non-inoculated with *S. sclerotiorum*. The antifungal effect was measured based on a disease index. The photosynthetic and growth parameters of NF-treated plants were evaluated. In addition, the quantum translocation of NFs in plants was examined, using Alizarin Red-labeled NFs, and their detection was monitored under fluorescent microscopy.

## 2. Materials and Methods

Chemicals and Reagents. All the reagents were of analytical grade and were used without any further purification: Copper(II) nitrate trihydrate (Cu(NO_3_)_2_·3H_2_O) (Merck, ≥99.5%, *M* = 241.60 g/mol), zinc(II) nitrate tetrahydrate (Zn(NO_3_)_2_·4H_2_O) (Merck, ≥99.5%, M = 261.44 g/mol), zinc acetylacetonate hydrate (Zn(acac)_2_) (Aldrich, *M* = 263.61 g/mol), diethylene glycol (DEG) (Merck, ≥99%, *M* = 106.12 g/mol), polyethylene glycol 8000 (PEG 8000) (Merck, ≥99%), KOCIDE 2000 35 WG (K&N Efthymiadis agrochemicals, a.i. copper hydroxide 35% *w*/*w*), potato dextrose agar (PDA) (Merck), and Alizarin Red S (C_14_H_7_NaO_7_S) (Sigma-Aldrich).

### 2.1. Solvothermal Synthesis of Bimetallic CuZn and Metal Oxide ZnO NFs

A modified polyol process under solvothermal conditions was used for the synthesis of INPs as a cost-effective and reproducible method. To isolate the specific compositions, reaction conditions were selected based on our previous reports [28,29].

*Bimetallic CuZn@DEG NFs*. Cu(NO_3_)_2_·3H_2_O (1 mmol) and Zn(NO_3_)_2_·4H_2_O (1 mmol) were mixed and dissolved in 30 mL of diethylene glycol (DEG) under vigorous stirring at 35 °C and then transferred into a Teflon sealed vessel. The reaction was carried out at 220 °C with a hold time of 8 h and ramp time heating step (from 25 to 220 °C) set at 45 min. The supernatant liquid was separated by centrifugation at 5000 rpm for 20 min and discarded. A brown precipitate was obtained after washing with ethanol at least three times to remove the excess of ligands and the unreacted precursors. A yield of 56% was based on metal precursors.

*ZnO@PEG8000 NFs*. Zn(acac)_2_ (1.06 mmol) and polyethylene glycol 8000 (0.11 mmol) were mixed and dissolved with vigorous stirring at 35 °C. The solution was transferred into a Teflon sealed vessel. The reaction was carried out at 220 °C with a hold time of 12 h and ramp time heating step (from 25 to 220 °C) set at 45 min. After centrifugation at 5000 rpm for 20 min, the supernatant liquid was discarded, the precipitate was washed three times with ethanol to dispose of any by-products, and a light-brown precipitate was obtained. The yield was 45% based on the metal precursor.

### 2.2. Characterization

The crystal structure and crystallite size of synthesized NFs were obtained through Bragg–Brentano (BB) geometry using a two-cycle Rigaku Ultima+ powder X-ray diffractometer. Scans were taken with a 2θ step size of 0.01 °C with Cu-Ka (40 kV/30 mA) radiations in the 2θ range from 10° to 80°. TGA measurements were recorded under a flow of N_2_. The temperature was raised from room temperature up to 800 °C at a heating rate of 10 °C/min. TEM observations were carried out on a JEOL JEM 1200-EX transmission electron microscope at 120 kV. For the preparation of samples, a drop of dilute NFs colloidal suspension was placed onto a carbon-coated copper grid and left to dry at ambient temperature. The bonds and the chemical information of the NFs were determined by FT-IR spectra (Nicolet 6700 FT-IR Spectrometer) in the wavenumber range of 4000–400 cm^−1^ (2 cm^−1^ resolutions; 30 scans). The hydrodynamic size and *ζ*-potential measurements were performed at room temperature with a Nano ZS Malvern apparatus.

### 2.3. Ionic Release Measurements

The ionic release of copper and zinc ions from the CuZn@DEG and ZnO@PEG NFs into distilled water (DI) was studied by preparing a solution of 10 mg of NFs in 100 mL of DI. The solution was sonicated for 10 min, and then, the residual Cu^2+^ and Zn^2+^ concentration was determined after 24, 48, 72, and 96 h utilizing the Inductively Coupled Plasma Atomic Emission Spectroscopy (ICP-AES) analysis (iCap 6300, Thermo Scientific).

### 2.4. Antifungal Bioassays

#### 2.4.1. Strains and Cultural Practice

Two pathogenic fungi, *B. cinerea* and *S. sclerotiorum*, were obtained from the culture collection of the Laboratory of Plant Pathology, School of Agriculture, Faculty of Agriculture, Forestry and Natural Environment, Aristotle University of Thessaloniki. Stock cultures of the fungi were cultured on PDA at 25 °C in the dark.

##### *In Vitro* Antifungal Activity

Antifungal activity was investigated using the agar dilution method according to Banik and Perez-de-Luque, with slight modifications [24]. The PDA medium was autoclaved, and the calculated amounts of NFs were added when its temperature reached 40 °C without prior dilution. NFs have been also pre-sterilized at 120 °C, as their structure is stable up to 350 °C (based on the TGA results). The NFs were weighed to achieve concentrations of 50, 100, 200, 400, 600, 800, 1000, 1200, and 1400 μg/mL, added to the sterile PDA, and placed in an ultrasonic bath (Transsonic 460 h, Elma GmbH & Co KG, Germany) for 60 s to achieve a uniform dispersion of the nanoparticles. These mixtures were poured into Petri dishes (6 cm diameter), and the fungi were inoculated after solidification, as a disc of 0.5 cm of fungal material, transported from a 7-day-old culture. Dishes containing PDA without any NFs were used as a control, and dishes containing a commercial formulation of copper hydroxide (KOCIDE 2000 35 WG at the highest recommended dose) were used as a chemical control. DEG and PEG 8000 were also tested separately in order to exclude any possible inhibition of the fungi caused by those materials. The fungal plugs were placed in the center of the Petri dishes and incubated at 25 °C until the control Petri dishes were totally covered by the fungus.

Evaluation of antifungal activity was implemented daily by measuring the diameter of *B. cinerea* and *S. sclerotinia* colonies. Fungal growth inhibition was calculated according to the formula:

$$Inhibition\;\left(\%\right)=\frac{\mathrm{dc}-\mathrm{dt}}{\mathrm{dc}}\times 100\;\;$$

where dc is the average diameter of linear growth in control, and dt is the average diameter of linear growth in treatment. All bioassays were performed in five replicates, and the experiment was repeated twice.

Mean EC_50_ values (half-maximal effective concentration causing 50% inhibition of mycelial growth) of ZnO@PEG NFs and bimetallic CuZn@DEG NFs against *B. cinerea* and *S. sclerotiorum* were calculated using graded dose–response curves.

##### Plant Material and Growth Conditions

*Lactuca sativa* (Lettuce, *Romana*, *Green Towers MI*, 7 cm in height) plants were kindly provided by AGRIS and transferred to a growth chamber with a 10 h photoperiod, 20 ± 1/18 ± 1 °C day/night temperature, photosynthetic photon flux density (PPFD) 130 ± 20 μmol quanta m^−2^ s^−1^, and relative humidity 50 ± 5/60 ± 5% day/night.

##### *In Planta* Experiments

Lettuce—*S. sclerotiorum* pathosystem: Ten colonized wheat grains were used as inoculum, as described by Taylor et al. [30]. Lettuce stems were first cut at four different places using a scalpel, and ten wheat grains were placed around and right next to the wounded stems (Figure S1). In addition to the non-treated control, injured plants without any treatment were also evaluated in order to exclude any wilting symptoms attributed to the wounds of the stem. Plants treated with KOCIDE 2000 35 WG at the highest recommended dose were used as a chemical control. After an incubation period of 48 h in the growth chamber where humidity was maintained at a high level (65 ± 5/70 ± 5% day/night %), plants were treated with NFs at concentrations causing 100% inhibition of *S. sclerotiorum* based on the EC_50_ values. Evaluation of the progressing symptoms and the fungal inhibition ability of the NPs was performed every 24 h for 72 h. The disease index was determined according to Huzar and Dorrance [31], with minor modifications: (1) no symptoms—healthy plants, (2) stem infection extended for 3–5 mm, (3) stem infection > 5 mm, (4) infection progressing to the leaves, (5) lesions appearance on the stem, and (6) wilted plant. The bioassay included twelve replications per treatment, and the experiment was repeated twice.

### 2.5. Measurement of Photosynthetic Parameters

The photosynthetic aspects were determined 48 h after treatment with NFs and chemical fungicide KOCIDE 2000 (i) in lettuce inoculated with *S. sclerotiorum* and (ii) in lettuce non-inoculated with fungi. Measurements of net photosynthesis (A_net_, μmol CO_2_ m^−2^s^−1^) were performed during the time interval 10 a.m. to 12 p.m. on fully expanded lettuce leaves for each treatment, using the open-flow portable photosynthesis systems (LCPROT-001/BW, Advanced Portable Photosynthesis System, ADC Bioscientific Ltd., Hoddesdon, UK.). The photosynthetic quantum yield (QY) or photosynthetic efficiency was recorded by a Portable Fluorometer (FluorPen FP 100). The chlorophyll content index (CCI) was measured using an Opti-Sciences CCM-200 chlorophyll meter. Five replications per treatment were used for each measurement.

### 2.6. Florescence Detection of NFs in Treated Lettuce Plants

In order to evaluate the uptake and the translocation of NFs in treated plants, the synthesized CuZn@DEG and ZnO@PEG NFs were dyed with Alizarin Red-S based on our recent study by Gkanatsiou et al. [32]. Lettuce plants inoculated with *S. sclerotiorum* were sprayed with (i) a solution of Alizarine Red S and water, (ii) labeled CuZn@DEG NFs with Alizarin Red S, and (iii) labeled ZnO@PEG NFs with Alizarin Red S at the same concentrations as in the *in planta* experiments. The presence of NFs in different parts of lettuce plants (leaves, stems, and roots) after 48 h of foliar application was observed with a Florescence Microscope 100–240 V, 75 VA, 50/60 Hz (OPTICA^®^, Ponteranica-Italy) at the emission value of the fluorescent dye around 600 nm.

### 2.7. Statistical Analysis

The EC_50_ values, the NFs concentration that inhibits the fungus growth by 50%, were calculated using a non-linear dose–response curve through Origin Pro 8 (Data Analysis and Graphing Software) and after five replicates per NFs concentration. Data from the *in planta* experiments were analyzed by analysis of variance (ANOVA), based on the completely randomized design (CRD), and mean values were computed from the respective replicates. Comparisons were made using Tukey’s test at a significance level of *p* ≤ 0.05. All statistical analyses were performed with the SPSS v 25.0 software (SPSS Inc. Chicago, IL, USA).

## 3. Results and Discussion

### 3.1. Synthetic Aspects and Characterization

Several factors are responsible for the uptake of INPs by plants. The size, shape, and organic coating influence the rate of dissolution and bioactivity, and they are considered important factors affecting the efficiency of the INPs in bioassays [33,34]. Coated INPs have gained excessive interest, as interfacial effects govern the properties of both organic and inorganic components of these hybrid structures. Among several organic ligands, we have shown previously that polyols can be utilized in a triple role (solvent, stabilizing, and reducing agent) and play a vital role in the size, shape, morphology, and stability regulation of the synthesized INPs while they are considered eco-friendly compounds [28,29,35]. The degree of INPs’ aggregation can be controlled; terminal hydroxyl groups give rise to hydrophilicity and as such can be used to direct INPs’ wet self-assembly chemical synthesis. Thus, DEG and PEG 8000, polyols of different chain length and physicochemical properties, have been used in a triple role in the course of solvothermal synthesis to isolate CuZn@DEG and ZnO@PEG NFs, respectively.

**Figure 1:**
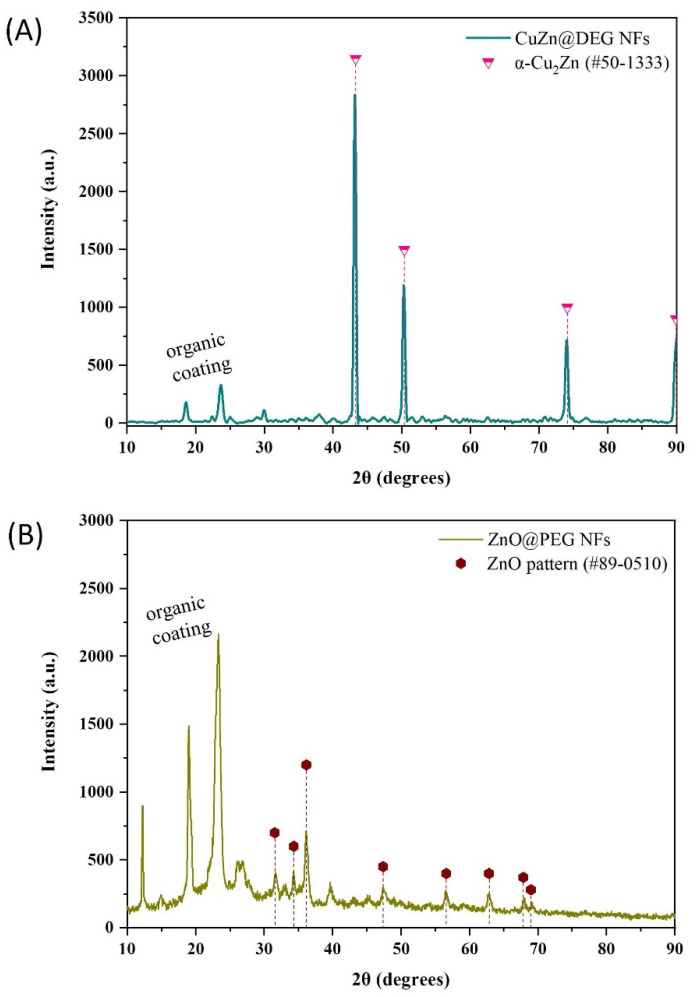
XRD measurements of bimetallic CuZn@DEG (**A**) and metal oxide ZnO@PEG (**B**) nanoflowers.

The XRD pattern of synthesized bimetallic CuZn@DEG is illustrated in Figure 1A. The peaks at 43.3°, 50.5°, and 74.2° were attributed to the planes (111), (200), and (220) of Cu_3_Zn α-brass (#50-1313, space group Fm-3m) [36]. The sharp diffraction peaks confirm the good crystallinity of the synthesized INPs. The crystallite size was calculated according to the Debye’s Scherer equation using the full width at half-maximum (FWHM) data to be about 40 nm. The lattice parameter (a = b = c in the fcc lattice) values were estimated at 3.615 Å. The quantitative analysis calculated by using MDI’s Jade software found *α*-Cu_67_Zn_33_ (α-brass phase) [28]. Additionally, the composition of CuZn@DEG NFs was estimated via SEM/EDX analysis (Figure S2, Table S1) at Cu_0.71_Zn_0.29_ very close to the quantitative results by XRD calculation and in agreement with the α-brass fcc phase. Figure 1B shows the XRD pattern of ZnO NPs. The diffraction peaks located at 31.6°, 34.3°, 36.2°, 47.4°, 56.7°, 62.9°, 67.9°, and 68.7° were indexed to the hexagonal wurtzite phase (#89-0510, space group P63mc (186)) and correspond to reflections from (1 0 0), (0 0 2), (1 0 1), (1 0 2), (1 1 0), (1 0 3), (1 1 2), and (2 0 1) planes, respectively [37]. The crystallite size of ZnO@PEG was found to be 15 nm, which is derived from the FWHM of more intense peaks corresponding to the (101) plane located at 36.2°. The major peaks at 2θ = 14.8°, 19.1°, and 23.4° and a few minor peaks at 26.2°, 26.8°, 35.1°, 39.7°, and 45° correspond to the crystallized organic coating PEG 8000 and are consistent with previous reports [38].

**Figure 2:**
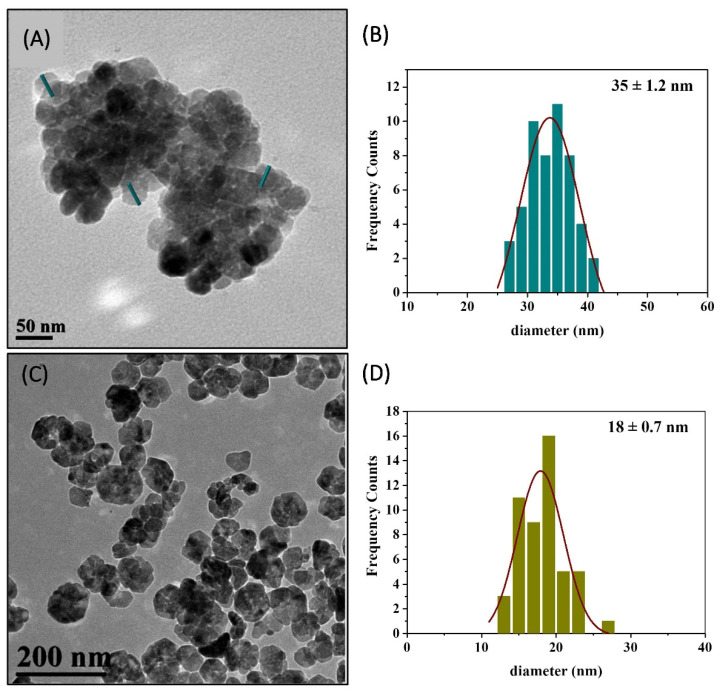
TEM image of bimetallic CuZn@DEG NFs (**A**), histogram with average size (±SD) of CuZn@DEG NFs (**B**), TEM image of ZnO@PEG NFs (**C**), and histogram with average size (±SD) of ZnO@PEG NFs (**D**). SD: Standard Deviation.

TEM images of the bimetallic CuZn@DEG showed a nanoflower (NF) morphology (Figure 2A) with a diameter of 172 nm. These flower-like structures consist of nanoparticles with an average size of 35 ± 1.2 nm (Figure 2B), which is in line with the estimated crystallite size obtained by XRD. The TEM image of ZnO@PEG displays also NF structures (Figure 2C), of smaller size, 69 nm (Figure 2D), that were formed through the assembly of hexagonal nanoparticles with an average diameter of 18.1 ± 0.7 nm. ZnO flower-like structures through different synthetic processes and with larger crystalline sizes have been reported previously with average crystallite sizes of 21.9 nm [39], about 30.8 to 31.6 nm [40], 30–70 nm [41] and 44.2 nm [42].

**Figure 3:**
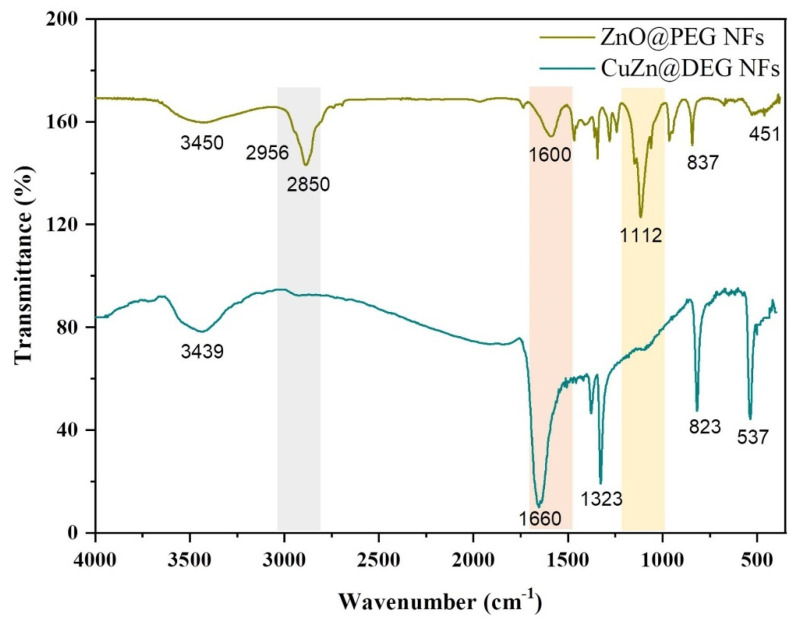
FTIR spectra of CuZn@DEG and ZnO@PEG NFs in the wavenumber region of 4000–400 cm^−1^.

The presence of the organic coating on the surface of the NFs has been confirmed by FTIR spectra (Figure 3). The common nature of the polyols that have been used as coating to the NPs surface is indicative in both cases. The broad band at about 3450 cm^−1^ is assigned to the –OH stretching mode of the glycol molecules and/or absorbed water. CuZn@DEG displays the sharpest peak in this region of the spectra compared with ZnO@PEG, owing to free hydroxyl groups on the surface of the NPs. Peaks at ≈2900 and ≈2850 cm^−1^ confirm the asymmetric and symmetric stretch of the –CH_2_ groups of PEG for ZnO@PEG. The strong absorption peak at 1660 cm^−1^ and the medium peak at 1600 cm^−1^ corresponded to the oxidized form of DEG and PEG, respectively. Oxidized forms of polyols coming from one end and/or two end such as aldehydic, ketonic, carboxylic groups are attributed to solvothermal synthetic conditions [10,35]. DEG exhibits the strongest reductive ability and is prone to oxidation compared to PEG 8000. The triple strong peak centered at 1112 cm^−1^ matches well with the characteristic CH_2_−O−CH_2_ rocking mode of polymeric polyol PEG 8000. Finally, no clear peaks appear in the region of the spectra below 500 cm^−1^, confirming the metallic nature of the as-synthesized CuZn@DEG, while the peak at 451 cm^−1^ is attributed to the Zn−O stretching vibration mode and is consistent with previous reports [43].

**Figure 4:**
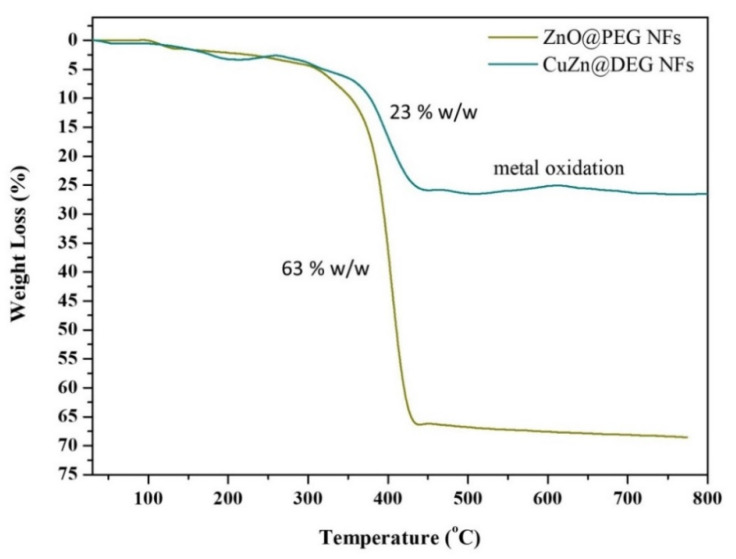
Thermogravimetric analysis curves of bimetallic CuZn@DEG and metal oxide ZnO@PEG NFs in the range of 30–800 °C.

TGA in the temperature range of 30 to 800 °C was conducted to calculate the amount of the organic coating on the surface of the produced CuZn@DEG and ZnO@PEG NFs, and these measurements are illustrated at Figure 4. The cumulative organic contents were 23 and 63 wt %, for CuZn@DEG and ZnO@PEG NFs, respectively. For both samples, about 3% weight loss up to 200 °C is attributed to the physically adsorbed water molecules on the surface of the NPs. The decomposition of the coatings begins around 350 °C and is completed just above 450 °C. In addition, for the bimetallic CuZn@DEG NFs, at 600 °C, metal oxidation occurs, which is indicative of the metallic nature of the NFs.

The colloidal stability of the NFs in water is an important issue especially for agro-applications. DLS of aqueous dispersions of CuZn@DEG and ZnO@PEG NFs was carried out to investigate their hydrodynamic diameter and ζ-potential. It has to be mentioned that dried samples could be readily redispersed in water, without any further modification, which is indicative of their hydrophilicity. The hydrodynamic size was determined at 306 and 338 nm for CuZn@DEG and ZnO@PEG NFs, respectively (Figure S3). The increased hydrodynamic sizes in comparison with the sizes by TEM can be regarded because of the large organic content and swelling of PEG in water, while agglomeration/extended networks formation cannot be excluded [44]. Additionally, the ζ-potential values of the aqueous suspensions of CuZn@DEG and ZnO@PEG NFs were found to be −16.5 mV and + 2.5 mV, respectively (Figure S4). The colloidal stabilization is attributed to the steric hindrance effect and/or electrostatic repulsion that are coming from the coating of DEG and PEG in accordance with other studies e.g., [45]. The physicochemical characteristics of NFs are summarized in Table 1.

The ionic release (Cu^2+^ and Zn^2+^ ions) was measured in DI water through leaching tests in aqueous solutions to determine the stability of NFs after 24, 48, 72, and 96 h (Table 2). The concentration of Zn^2+^ ions released in water was increased from 1.03 to 3.75 μg/mL after a 96 h dispersion of ZnO@PEG NFs (100 μg/mL) in DI. The slow release and the low rate of Zn^2+^ ions concentration were expected, since the presence of a high amount of polyol coating (63% *w*/*w* PEG 8000) in surface makes them more stable. Surface coatings can affect the dissolution or release of the ions from NFs. On the other side, the concentration of Cu^2+^ ions released from CuZn@DEG NFs was increased from 4.42 to 6.51 μg/mL, while the concentration of Zn^2+^ ions was increased from 13.17 to 16.03 μg/mL after 96 h. In case of CuZn@DEG NFs, a higher rate of Zn^2+^ ions release was observed compared to that from ZnO@PEG NFs attributed to the lower amount of polyol coating (23% *w*/*w* DEG). In addition, the reason of less Cu^2+^ ions leaching instead of Zn^2+^ ions, in case of CuZn@DEG NFs, is attributed to the random arrangement of Cu and Zn atoms in the alloy structure, suggesting that the Zn atoms are located on the surface of the fcc unit cell. The leaching of Cu^2+^ from the NPs occurs to some degree naturally and depends on their size [46]. However, in both cases, ionic release can be considered low, as NFs provide a slow ionic release tendency over time.

### 3.2. In Vitro Antifungal Activity

**Figure 5:**
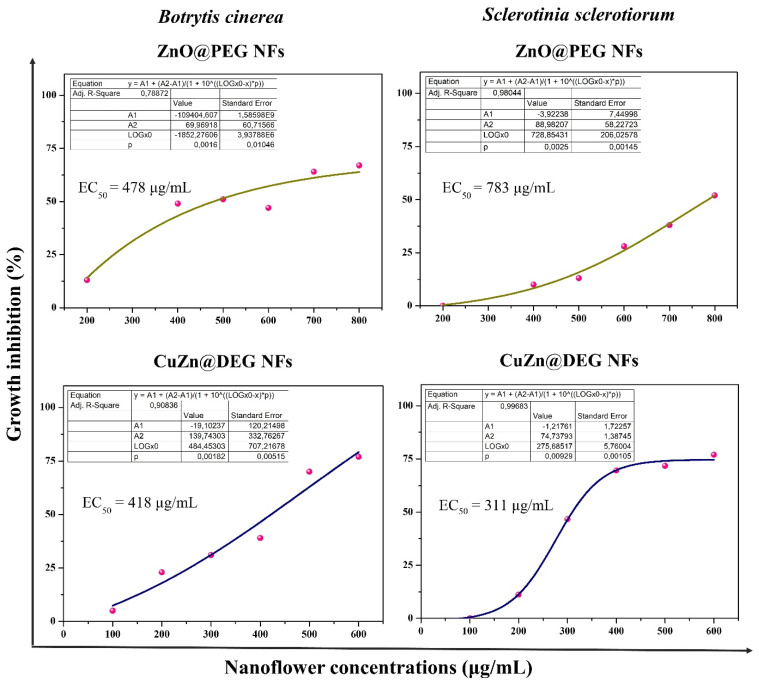
Dose-response growth inhibition curves. Growth inhibition (%) of the fungi *B. cinerea* and *S. sclerotiorum* exposed to different concentrations of ZnO@PEG and CuZn@DEG NFs. Each point represents the mean of five replicates per NFs concentration (two experiments’ replications).

The *in vitro* evaluation of antifungal activity of ZnO@PEG and CuZn@DEG NFs against *B. cinerea* and *S. sclerotiorum* was demonstrated. The antifungal action of NFs was estimated in terms of percent inhibition of mycelial growth, and the EC_50_ values were calculated from the corresponding curves (Figure 5). Clear time and dose–response relationships were established for ZnO@PEG and CuZn@DEG NFs, showing that both NFs inhibit the growth of fungal strains (Figure S5). DEG and PEG 8000 were also studied and showed no inhibition against either of the fungi. The mean EC_50_ values of *B. cinerea* caused by ZnO@PEG and CuZn@DEG NFs were calculated at 478 μg/mL and 418 μg/mL, respectively, after 96 h. Meanwhile, the EC_50_ values of *S. sclerotiorum* to ZnO@PEG and CuZn@DEG NFs were calculated at 783 μg/mL and 311 μg/mL, respectively, after 72 h. Apart from the inherent tolerance of fungus, the lower efficiency of the ZnO@PEG in comparison with CuZn@DEG NFs in both fungi is mainly attributed to the composition effect and/or metal leaching dissimilarities, taking into account the similar hydrodynamic sizes of the NFs. ZnO@PEG NFs are found to be more effective than the commercial naked ZnO NPs with an approximate size of <50 nm, which showed EC_50_ = 670 μg/mL against *B. cinerea* [47]. However, commercial naked rod-like ZnO NPs (70 ± 15 nm) significantly inhibited the growth of *B. cinerea* at 244 μg/mL [48]. The growth inhibition depends on the shape of the particle [49], which means that the interaction between the particle and the fungi is selective, which is possibly due to the nature of the crystalline-exposed surface present in the contact faces with the *B. cinerea*. Moreover, the effectiveness depends on the surface coating and its polymer chain lengths, since the percentage (% *w*/*w*) of PEG 8000 in case of ZnO@PEG NFs is 63% and has a long chain. Regarding *S. sclerotiorum*, even though it is an important plant fungal pathogen with a broad host range [50], limited information was found. Abdel-Halim and El-Ghanam report a 73.13% fungal inhibition exhibited by spherical ZnO NPs (20–40 nm) at a concentration of 1000 μg/mL [51]. This percentage of inhibition is considered approximately equal to the effectiveness of the present flower-like ZnO@PEG NPs (15 nm), demonstrating the importance of size similarity in antifungal activity. Meanwhile, ZnO NPs with a morphology similar to a fan and bouquet structure (27–40 nm width; 134–200 nm length) tended to reduce the mycelial growth of *S. sclerotiorum* at 5, 10, nd 20 μg/mL, but these results were not statistically significant [52].

### 3.3. In Planta Experiments

#### 3.3.1. Disease Severity

**Figure 6:**
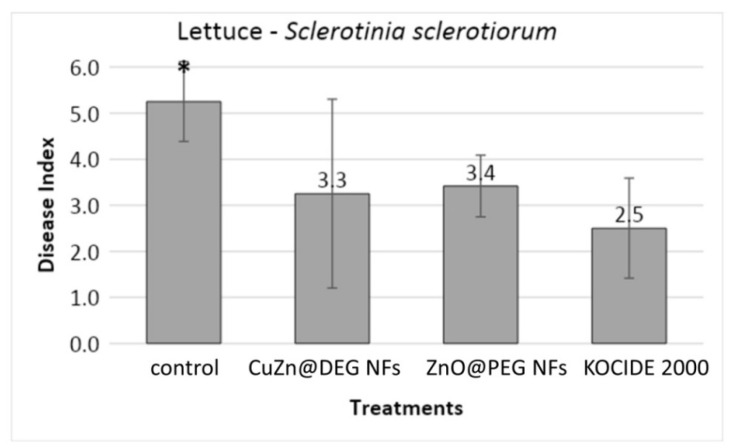
Effect of CuZn@DEG and ZnO@PEG NFs on disease severity caused by *S. sclerotiorum* on lettuce plants. Control plants were treated only with the pathogen, and KOCIDE 2000 (35 WG) was used as a chemical control. Error bars represent the standard deviation based on twelve technical replicates. An asterisk indicates a statistically significant difference according to Tukey’s test (*p* ≤ 0.05).

**Figure 7:**
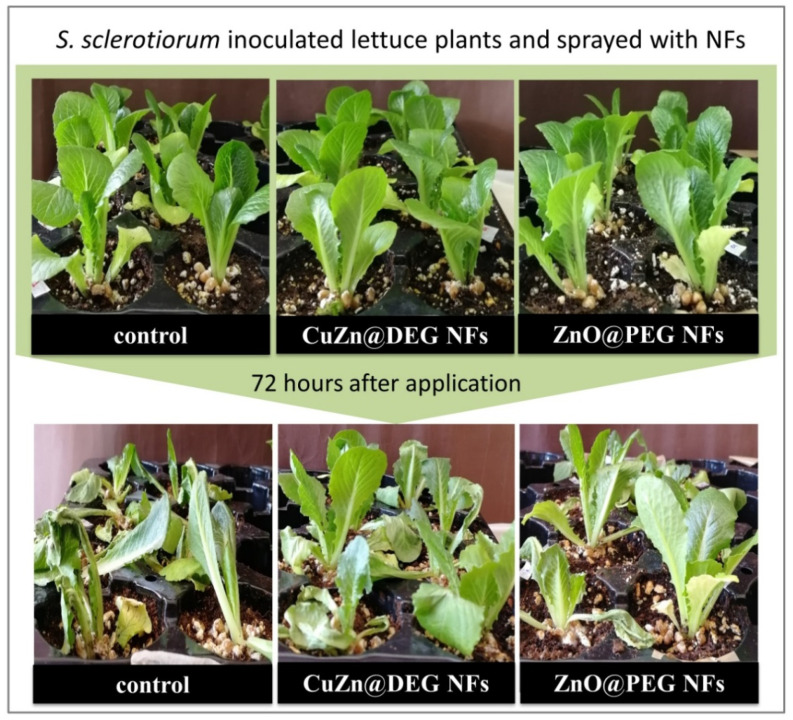
Lettuce plants inoculated by *S. sclerotiorum* and treated with CuZn@DEG and ZnO@PEG NFs at 0 h and 72 h after application.

The antifungal activity was confirmed by estimating the disease severity in lettuce plants as described above. Lettuce stems were injured and framed with wheat grains inoculated with *S. sclerotiorum*, and then, leaves were sprayed with NFs and chemical control. The commercial widely used chemical fungicide KOCIDE 2000 (copper hydroxide) was selected and utilized as a positive control. Based on the *in vitro* study, the foliar application of ZnO@PEG and CuZn@DEG NFs was performed at concentrations causing 100% inhibition of *S. sclerotiorum*, while KOCIDE 2000 was used at the highest recommended dose. Evaluation of the progressing symptoms and the fungal inhibition ability of the NFs was performed every 24 h for 72 h. Results indicated that both NFs treatments exhibit a decrease in disease index and were statistically not different compared to the chemical control, as shown in Figure 6. No phytotoxic effects were observed on lettuce plants sprayed with NFs and KOCIDE 2000. Figure 7 illustrates lettuce plants inoculated with *S. sclerotiorum* and sprayed with NFs 72 h after application. In the case of control, extensive symptoms are visible on the lower stems and upper leaves of infected lettuce; while regarding the plants sprayed with NFs, the limitation of fungal growth is obvious, proving the effectiveness of the NFs.

Metal ions such as Zn^2+^ and Cu^2+^ are considered as a stimulating force behind the antifungal properties [53] that might be moved inside the fungal cells or attached to their outer surfaces, resulting in cell apoptosis via protein denaturation and disruption of the cell membrane [54]. Thus, ZnO@PEG and CuZn@DEG NFs inhibit fungal growth and reduce the symptoms’ intensity. They could be proposed as promising fungicides with non-phytotoxic effects in plants as part of an integrated fungal disease strategy. Recently, the activity of ZnO NPs and ZnO bulk against *S. sclerotiorum* on eggplant (*Solanum melongena* L.) was investigated, where the half-maximal inhibitory concentrations (IC_50_) were found at 4150 and 6280 μg/mL, respectively [55]. The antifungal action of metallic NPs was attributed to the prevention of the development of conidiophores and conidia of *S. sclerotiorum*, which eventually led to the death of fungal hyphae. Additionally, the foliar application of ZnO NPs and their exposure under the light resulted in an improvement in the antimycotic effects [56]. Therefore, the photon-induced generation of reactive oxygen species (ROS) and a poisoning effect due to Zn^2+^ release are the two main contributors to the antifungal activity of ZnO NPs [57].

#### 3.3.2. Photosynthetic Characteristics

The photosynthetic characteristics of the lettuce plants that were inoculated with *S. sclerotiorum* and sprayed with CuZn@DEG, ZnO@PEG NFs, and KOCIDE 2000 were measured. The Anet, CCI, and QY values 72 h after treatment are presented in Table 3. In addition, the photosynthetic characteristics of non-inoculated lettuce plants were demonstrated, and the Anet value of ZnO@DEG NFs was observed to have a statistically significant difference compared to that of the control (data not shown). The A_net_ and the QY values of inoculated lettuce sprayed with ZnO@PEG NFs is 4.70 ± 0.34 and 0.72 ± 0.012, respectively, and they are higher and statistically different (*p* ≤ 0.05) compared to CuZn@DEG NFs and KOCIDE 2000 treatments. The CCI rate for both NFs was found to be statistically similar but statistically different from the control. In all cases, no phytotoxicity was observed in the lettuce plants after treatment with the NFs as well as the chemical control. Generally, ZnO as a semiconductor exhibits high photocatalytic activity that plays a major role in the antifungal and photosynthetic efficiency. The application of ZnO NPs in tomato plants enhanced the photosynthetic rate by upgrading the activity of enzymes and boosting proline concentration [58]. The foliar exposure of wheat (*Triticum aestivum* L.) to ZnO NPs boosted up the leaf chlorophyll contents and also decreased the oxidative stress and enhanced the leaf superoxide dismutase and peroxidase activities compared to the control [59].

Metal oxide NPs, such as ZnO, affect the energy transfer efficiency of isolated photosynthetic reaction centers, as well as photochemical fluorescence, and increase the quantum yield in plants [60]. Once the plants are able to absorb and utilize sun energy, ZnO NPs work as photocatalysts, enhancing the conversion of sun light into chemical energy. This photocatalytic activity is variant in different NPs and depends on their morphology [61]. Rossi et al. found that *Coffea arabica* sprayed with ZnO NPs (10 mg/L) showed an increase in the A_net_ rate of about 55% compared to the control 45 days after treatment [62]. Moreover, Luksiene et al. reported that ZnO NPs in the presence of UV and visible light were activated and protect strawberries from *B. cinerea*, decreasing the spoilage of fruits. In contrast, when the same concentration of NPs was applied in the dark, the antifungal activity was significantly inferior [63]. Although there is evidence that copper fungicides may inhibit chlorophyll synthesis and net photosynthesis [64], the bimetallic agents used in the current study were not only very potent antifungal agents but also enhanced the photosynthetic parameters.

#### 3.3.3. Uptake and Translocation of NFs

**Figure 8:**
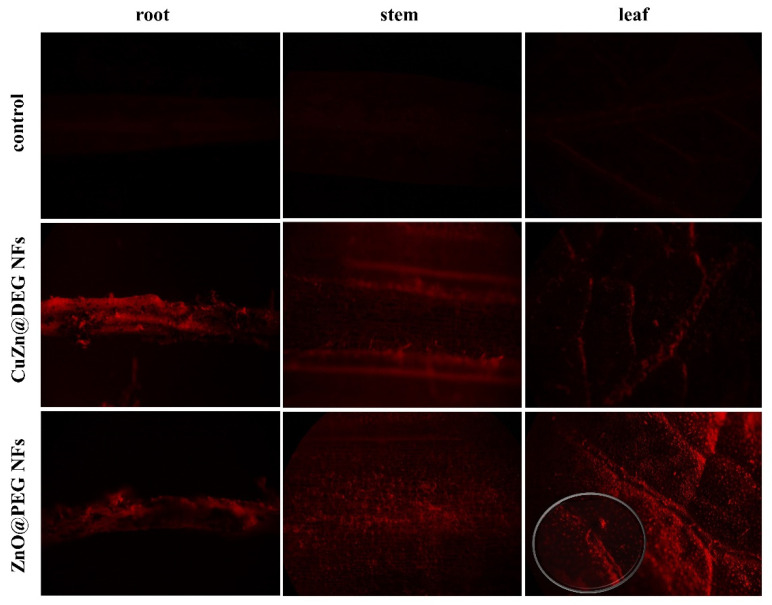
Florescence images of the root, stem, and leaf parts of lettuce plants inoculated by *S. sclerotiorum* and sprayed with Alizarin Red S labeled CuZn@DEG and ZnO@PEG NFs.

The translocation of the NFs in plant tissues was investigated using Alizarin Red S labeled NFs. In the current work, since the NFs were applied both foliar and by root, the presence of NFs in plants was monitored in leaves, stem, and roots. CuZn@DEG and ZnO@PEG NFs labeled with Alizarin Red S were sprayed on lettuce plants, and 72 h after treatment, the plants were inoculated with *S. sclerotiorum*. Lettuce plants sprayed only with Alizarin Red S were used as control. Figure 8 illustrates the fluorescence images of foliar application of NFs and infection of lettuce with *S. sclerotiorum*. In plant leaves sprayed with Alizarin Red S solution, no fluorescent signal was detected. Foliar exposure of labeled CuZn@DEG NFs showed the uptake from the leaf surface to the internal tissues, while the red florescence signal denotes the presence of aggregated NFs that penetrate with water, once they are hydrophilic. In case of ZnO@PEG NFs, it seems that most of the particles stay on leaves, although an amount of NFs was shown to be present on the stem and roots, with a lower fluorescent signal in parts of the root (Figure 8). The degree of aggregation and metal ionic release are considered the main factors for their transportation. The translocation from leaf to root is achieved via the stomata or entrapment of NPs in the cuticle, distributing into the stems and then into roots via phloem vessels [65]. PEGylated materials give stealth characteristics that can mimic the extracellular matrix environment and penetrate inside the cell. Previously, it has been shown that NPs were mainly trapped on the leaf surface [66], while other studies revealed aggregation in the root apoplastic space indicating the existence of a pathway for NPs in plant roots [67]. For example, ZnO NPs of 20 nm have been aggregated in ryegrass roots and CuO NPs (43 nm) have been aggregated in *Elsholtzia splendens* roots [44], while Dimkpa et al. indicated that the Zn uptake, translocation, and accumulation in the plant system take place in the form of Zn^2+^ ions that are released from NPs [68]. Although the detection of nanoparticles in different plant parts 72 h after application indicates translocation, the extent of probable bioaccumulation that may impact the food chain needs further study. Therefore, additional experimentation with respect to nanoparticle–plant interactions, and focusing on TEM pictures of different plant parts and cell types, such as guard cells, taken at different time intervals after NPs application, would elucidate possible bioaccumulation dynamics.

## 4. Conclusions

Modern agriculture is renovating into precision agriculture, and an increasing need to reduce the environmental impact of pesticides and to develop alternative agrochemicals is evident. The development of alternative fungi management strategies is mandatory, as the few available chemical fungicides have been excessively used over the last few decades with serious concerns on human health and the environment impact. Under this venue, bimetallic and metal oxide nanoflowers, CuZn@DEG and ZnO@PEG NFs readily dispersed in water, were solvothermally prepared in a facile one-step synthesis as novel nanostructures and have been examined as nano-fungicides. Additionally, the organic coatings of the NFs as polyol derivatives are biocompatible polymers that provide stealth characteristics to the NFs and are considered eco-friendly compounds. The hybrid organic/inorganic nanomaterials are readily dispersed in water, and no post-synthetic transformations are required. CuZn@DEG and ZnO@PEG NFs were evaluated regarding their inhibitory ability against *B. cinerea* and *S. sclerotiorum in vitro*. A significant growth inhibition of both fungi by ZnO@PEG and CuZn@DEG NFs in a dose-dependent manner was evident. To the best of our knowledge, this is the first report of NFs tested to control phytopathogenic fungi. Bimetallic CuZn@DEG NFs showed higher fungal growth inhibition compared to single metal oxide NFs (ZnO@PEG NFs), indicating a composition synergetic effect of the two bioessential metals (Cu/Zn) as the hydrodynamic sizes were found to be similar. Furthermore, the ability of ZnO@PEG and CuZn@DEG NFs to decrease the disease severity of White Rot (*Sclerotiniasis*) was confirmed on lettuce plants. Photosynthetic parameters, after application with ZnO@PEG NFs, showed higher values compared to all other treatments, indicating plant growth-enhancing ability. This effect is pronounced from the photoactive properties of ZnO as a semiconductor material that is activated under visible light. The translocation from leaves to roots through the stem was evident, while ZnO@PEG NFs were mainly trapped on the leaves. Flower-type structured materials pave the way for engineering new inorganic-based nano-fungicides, which could be included in an effective plant protection strategy and improve crop yield. Meanwhile, as each metal displays its own unique bioactivity, the potential synergistic action of bioessential metals in bimetallic structures could be investigated further to improve new evidence in plant protection. However, relatively little is still known about how NPs may affect plant life and get into the environment chain for a longer time span, and further evaluation is needed.

## Figures and Tables

**Table 1:** Summary of the physicochemical characteristics of the NFs.

Composition	Polyol Coating (% *w*/*w*)	d_XRDn_ (nm)	DLS (nm)	ζ-Potential (mV)
CuZn@DEG NFs	23	35 ± 1.2	306	−16.5
ZnO@PEG NFs	63	18 ± 0.7	338	+2.5

**Table 2:** Concentration (μg/mL) of Cu^2+^ and Zn^2+^ after 24, 48, 72, and 96 h dispersion of NFs (100 μg/mL) in distilled water.

	CuZn@DEG	NFs	ZnO@PEG NFs
Time (h)	Cu^2+^	Zn^2+^	Zn^2+^
24	4.41	13.17	1.03
48	4.56	13.30	2.99
72	5.31	14.74	3.06
96	6.51	16.03	3.75

**Table 3:** Effect of NFs and KOCIDE 2000 (35 WG) on the photosynthetic characteristics (Anet, CCI and QY) in leaves of *L. sativa* measured 72 h after foliar application.

Treatment	A_net_ (Mean ± SD)	CCI (Mean ± SD)	QY (Mean ± SD)
Control	2.99 ± 0.67 ^c^	7.66 ± 0.58 ^c^	0.71 ± 0.013 ^b^
CuZn@DEG NFs	3.00 ± 0.34 ^c^	11.12 ± 1.81 ^a^	0.68 ± 0.052 ^b^
ZnO@DEG NFs	4.70 ± 0.34 ^a^	11.46 ± 1.10 ^a^	0.72 ± 0.012 ^a^
KOCIDE 2000	3.88 ± 0.28 ^b^	9.82 ± 1.63 ^b^	0.69 ± 0.030 ^b^

Different letters (a, b, c) in each column indicate significant differences according to Tukey’s HSD post hoc test (*p* ≤ 0.05). Data are shown as mean ± SD. SD: Standard Deviation of five repeats per treatment.

## Supplementary Materials

The following are available online at https://www.mdpi.com/article/10.3390/ma14247600/s1, for Figures S1–S5 and Tables S1 please refer there.

## Abbreviations

A_net_	Net photosynthetic rate
*B. cinerea*	*Botrytis cinerea*
CCI	Chlorophyll content index
DEG	Diethylene glycol
DI water	Distilled Water
DLS	Dynamic Light Scattering
EC_50_	Values: Half maximal effective concentration causing 50% inhibition of mycelial growth
EDX	Energy Dispersive X-ray Analysis
FT-IR	Fourier transform infrared spectroscopy
FWHM	Full width at half-maximum
IC_50_	Half-maximal inhibitory concentration
ICP	Ionic Release Analysis
INPs	Inorganic nanoparticles
NFs	Nanoflowers
NPs	Nanoparticles
PDA	Potato dextrose agar
PEG 8000	Polyethylene glycol 8000
QY	Quantum yield
ROS	Reactive oxygen species
*S. sclerotiorum*	*Sclerotinia sclerotiorum*
SEM	Scanning electron microscope
TEM	Transmission electron microscopy
TGA	Thermogravimetric analysis
XRD	X-ray diffraction
ZnO NPs	Zinc oxide nanoparticles
USEPA	United States Environmental Protection Agency. Copper facts.

## References

[1] Dhiman S., Varma A., Goel A. (2021). Biofabricated nanoscale ZnO and their prospective in disease suppression and crop growth of Brassica species: A review. Biocatal. Agric. Biotechnol..

[2] Dean R., van Kan J.A.L., Pretorius Z.A., Hammond-Kosack K.E., di Pietro A., Spanu P.D., Rudd J.J., Dickman M., Kahmann R., Ellis J. (2012). The Top 10 Fungal Pathogens in Molecular Plant Pathology: Top 10 Fungal pathogens. Mol. Plant Pathol..

[3] Company P., González-Bosch C. (2003). Identification of a copper chaperone from tomato fruits infected with *Botrytis cinerea* by differential display. Biochem. Biophys. Res. Commun..

[4] Young C.S., Clarkson J.P., Smith J.A., Watling M., Phelps K., Whipps J.M. (2004). Environmental conditions influencing *Sclerotinia sclerotiorum* infection and disease development in lettuce. Plant Pathol..

[5] Adisa I.O., Pullagurala V.L.R., Peralta-Videa J.R., Dimkpa C.O., Elmer W.H., Gardea-Torresdey J.L., White J.C. (2019). Recent advances in nano-enabled fertilizers and pesticides: A critical review of mechanisms of action. Environ. Sci. Nano.

[6] Santiago E.F., Pontes M.S., Arruda G.J., Caires A.R.L., Colbeck I., Maldonado-Rodriguez R., Grillo R., Fraceto L.F., de Castro V.L.S.S., Grillo R., Ávila D., Oliveira H.C., Lima R. (2020). Understanding the Interaction of Nanopesticides with Plants. Nanopesticides.

[7] Giannousi K., Avramidis I., Dendrinou-Samara C. (2013). Synthesis, Characterization and Evaluation of Copper Based Nanoparticles as Agrochemicals against *Phytophthora infestans*. RSC Adv..

[8] Antonoglou O., Moustaka J., Adamakis S.I.-D., Sperdouli I., Pantazaki A.A., Moustakas M., Dendrinou-Samara C. (2018). Nano-brass CuZn Nanoparticles as Foliar Spray Nonphytotoxic Fungicides. ACS Appl. Mater. Interf..

[9] Sperdouli I., Moustaka J., Antonoglou O., Adamakis I.-D.S., Dendrinou-Samara C., Moustakas M. (2019). Leaf Age-Dependent Effects of Foliar-Sprayed CuZn Nanoparticles on Photosynthetic Efficiency and ROS Generation in *Arabidopsis thaliana*. Material.

[10] Tryfon P., Antonoglou O., Vourlias G., Mourdikoudis S., Menkissoglu-Spiroudi U., Dendrinou-Samara C. (2019). Tailoring Ca-Based Nanoparticles by Polyol Process for Use as Nematicidals and pH Adjusters in Agriculture. ACS Appl. Nano Mater..

[11] Oluwatoyin A.F., Ridwan O.A., Rizwan A.A. (2020). Nanoparticles’ Synthesis and Their Application in the Management of Phytonematodes: An Overview. Management of Phytonematodes: Recent Advances and Future Challenges.

[12] Iavicoli I., Leso V., Beezhold D., Shvedova A.A. (2017). Nanotechnology in agriculture: Opportunities, toxicological implications, and occupational risks. Toxicol. Appl. Pharmacol..

[13] Dimkpa O.C., Andrews J., Fugice J., Singh U., Bindraban S.P., Elmer H.W., Gardea-Torresdey L.J., White C.J. (2020). Facile Coating of Urea with Low-Dose ZnO Nanoparticles Promotes Wheat Performance and Enhances Zn Uptake Under Drought Stress. Front. Plant Sci..

[14] Mohamed A.A., Abu-Elghait M., Ahmed N.E., Salem S.S. (2021). Eco-friendly Mycogenic Synthesis of ZnO and CuO Nanoparticles for in Vitro Antibacterial, Antibiofilm, and Antifungal Applications. Biol. Trace Elem. Res..

[15] Abomuti M.A., Danish E.Y., Firoz A., Hasan N., Malik M.A. (2021). Green Synthesis of Zinc Oxide Nanoparticles Using *Salvia officinalis* Leaf Extract and Their Photocatalytic and Antifungal Activities. Biology.

[16] Rai M., Ingle A.P., Pandit R., Paralikar P., Sudhir Shende S., Gupta I., Biswas J.K., da Silva S.S. (2018). Copper and Copper Nanoparticles: Role in Management of Insect-pests and Pathogenic Microbes. Nanotechnol. Rev..

[17] Food and Drug Administration (FDA) Select Committee on GRAS Substances Opinion: Zinc Salts. USA. https://www.fda.gov/food/food-ingredients-packaging/food-ingredient-packaging-terms.

[18] Barman A. (2015). Review on Biocompatibility of ZnO Nano Particles. Resist. Train. Methods.

[19] Ali A., Phull A.-R., Zia M. (2018). Elemental zinc to zinc nanoparticles: Is ZnO NPs crucial for life? Synthesis, toxicological, and environmental concerns. Nanotechnol. Rev..

[20] Erazo A., Mosquera S.A., Rodríguez-Paéz J. (2019). Synthesis of ZnO nanoparticles with different morphology: Study of their antifungal effect on strains of *Aspergillus niger* and *Botrytis cinerea*. Mater. Chem. Phys..

[21] Sardella D., Gatt R., Valdramidis V.P. (2018). Assessing the efficacy of zinc oxide nanoparticles against *Penicillium expansum* by automated turbidimetric analysis. Mycology.

[22] Jamdagni P., Rana S.J., Khatri P., Nehra K. (2018). Comparative Account of Antifungal Activity of Green and Chemically Synthe-sized Zinc Oxide Nanoparticles in Combination with Agricultural Fungicides. Int. J. Nano Dimens..

[23] Kairyte K., Kadys A., Luksiene Z. (2013). Antibacterial and antifungal activity of photoactivated ZnO nanoparticles in suspension. J. Photochem. Photobiol. B Biol..

[24] Banik S., Luque A.P. (2017). In vitro Effects of Copper Nanoparticles on Plant Pathogens, Beneficial Microbes and Crop Plants. Span. J. Agricult. Res..

[25] Shende P., Kasture P., Gaud R. (2018). Nanoflowers: The future trend of nanotechnology for multi-applications. Artif. Cells Nanomed. Biotechnol..

[26] Castro A., Fernandes G.D.R., Franco O.L. (2014). Insights into novel antimicrobial compounds and antibiotic resistance genes from soil metagenomes. Front. Microbiol..

[27] Cartwright A., Jackson K., Morgan C., Anderson A.J., Britt D.W. (2020). A Review of Metal and Metal-Oxide Nanoparticle Coating Technologies to Inhibit Agglomeration and Increase Bioactivity for Agricultural Applications. Agronomy.

[28] Antonoglou O., Founta E., Karagkounis V., Pavlidou E., Litsardakis G., Mourdikoudis S., Thanh N.T.K., Dendrinou-Samara C. (2019). Structure Differentiation of Hydrophilic Brass Nanoparticles Using a Polyol Toolbox. Front. Chem..

[29] Giannousi K., Geromichalos G.D., Kakolyri D., Mourdikoudis S., Dendrinou-Samara C. (2020). Interaction of ZnO Nanostructures with Proteins: In Vitro Fibrillation/Antifibrillation Studies and in Silico Molecular Docking Simulations. ACS Chem. Neurosci..

[30] Taylor A., Coventry E., Handy C., West S.J., Young S.C., Clarkson P.J. (2018). Inoculum Potential of *Sclerotinia sclerotiorum sclerotia* Depends on Isolate and Host Plant. Plant Pathol..

[31] Huzar-Novakowiski J., Dorrance A.E. (2018). Ascospore Inoculum Density and Characterization of Components of Partial Resistance to *Sclerotinia sclerotiorum* in Soybean. Plant Dis..

[32] Gkanatsiou C., Karamanoli Κ., Menkissoglu-Spiroudi U., Dendrinou-Samara C. (2019). Composition Effect of Cu-based Nano-particles on Phytopathogenic Bacteria. Antibacterial Studies and Phytotoxicity Evaluation. Polyhedron.

[33] Wang Q., Zhang Y., Zheng J., Wang Y., Hu T., Meng C. (2017). Metal oxide decorated layered silicate magadiite for enhanced properties: Insight from ZnO and CuO decoration. Dalton Trans..

[34] Dimkpa O.C., Bindraban S.P. (2018). Nanofertilizers: New Products for the Industry?. J. Agric. Food Chem..

[35] Antonoglou O., Giannousi K., Arvanitidis J., Mourdikoudis S., Pantazaki A., Dendrinou-Samara C. (2017). Elucidation of one step synthesis of PEGylated CuFe bimetallic nanoparticles. Antimicrobial activity of CuFe@PEG vs Cu@PEG. J. Inorg. Biochem..

[36] Wang Z., Wang G., Louis C., Delannoy L. (2017). Novel Non-noble Bimetallic Cu-Zn/TiO_2_ Catalysts for Selective Hydrogenation of Butadiene. J. Catal..

[37] Ahammed R.K., Ashaduzzaman M., Paul C.S., Nath R.M., Bhowmik S., Saha O., Rahaman M.M., Bhowmik S., Aka T.D. (2020). Microwave Assisted Synthesis of Zinc Oxide (ZnO) Nanoparticles in a Noble Approach: Utilization for Antibacterial and Photocatalytic Activity. SN Appl. Sci..

[38] Jayaramudu T., Raghavendra M.G., Varaprasad K., Reddy S.V.G., Reddy B.A., Sudhakar K., Sadiku R.E. (2016). Preparation and Characterization of Poly(ethylene glycol) Stabilized Nano Silver Particles by a Mechanochemical Assisted Ball Mill Process. J. Appl. Polym. Sci..

[39] Anusiya A., Jansi B., Ravi G., Yuvakkumar R. Synthesis and Characterization of ZnO Nanoflowers. Proceedings of the International Conference on Momentous Role of Nanomaterials in Renewable Energy Devices.

[40] Qu Y., Huang R., Qi W., Shi M., Su R., He Z. (2020). Controllable synthesis of ZnO nanoflowers with structure-dependent photocatalytic activity. Catal. Today.

[41] Katiyar A., Kumar N., Shukla R.K., Srivastava A. (2020). Substrate Free Ultrasonic-assisted Hydrothermal Growth of ZnO Nanofowers at Low Temperature. SN Appl. Sci..

[42] Abdulgafour H., Hassan Z., Al-Hardan N., Yam F. (2010). Growth of zinc oxide nanoflowers by thermal evaporation method. Phys. B Condens. Matter.

[43] Nagaraju G., Udayabhanu S., Prashanth S.A., Shastri M., Yathisha K.V., Anupama C., Rangappa D. (2017). Electro-chemical Heavy Metal detection, Photocatalytic, Photoluminescence, Biodiesel Production and Antibacterial Activities of Ag–ZnO Nanomaterial. Mater. Res. Bull..

[44] Lin D., Xing B. (2008). Root Uptake and Phytotoxicity of ZnO Nanoparticles. Environ. Sci. Technol..

[45] Dong L., Li R., Wang L., Lan X., Sun H., Zhao Y., Wang L. (2021). Green synthesis of platinum nanoclusters using lentinan for sensitively colorimetric detection of glucose. Int. J. Biol. Macromol..

[46] Bondarenko O., Juganson K., Ivask A., Kasemets K., Monika Mortimer M., Kahru A. (2013). Toxicity of Ag, CuO and ZnO Na-noparticles to Selected Environmentally Relevant Test Organisms and Mammalian Cells *in vitro*: A Critical Review. Arch Toxicol..

[47] Malandrakis A.A., Kavroulakis N., Chrysikopoulos C. (2019). Use of copper, silver and zinc nanoparticles against foliar and soil-borne plant pathogens. Sci. Total. Environ..

[48] He L., Liu Y., Mustapha A., Lin M. (2011). Antifungal activity of zinc oxide nanoparticles against *Botrytis cinerea* and *Penicillium expansum*. Microbiol. Res..

[49] Pariona N., Mtz-Enriquez A.I., Sánchez-Rangel D., Carrión G., Paraguay-Delgado F., Rosas-Saito G. (2019). Green-synthesized copper nanoparticles as a potential antifungal against plant pathogens. RSC Adv..

[50] Jiang D., Fu Y., Guoqing L., Ghabrial S.A. (2013). Viruses of the Plant Pathogenic Fungus *Sclerotinia sclerotiorum*. Adv. Appl. Microbiol..

[51] Abdel-Halim K.Y., El-Ghanam A.A. (2019). Antifungal Potent of Some Metallic Nanoparticles against *Sclerotinia sclerotiorum* on Common Bean Plants: An Emphasis for Biochemical Alterations and Metal Accumulation. Acad. J. Life Sci..

[52] Consolo V.F., Torres-Nicolini A., Alvarez V.A. (2020). Mycosinthetized Ag, CuO and ZnO Nanoparticles from a Promising *Trichoderma harzianum* Strain and their Antifungal Potential Against Important Phytopathogens. Sci. Rep..

[53] Delgado K., Quijada R., Palma R., Palza H. (2011). Polypropylene with embedded copper metal or copper oxide nanoparticles as a novel plastic antimicrobial agent. Lett. Appl. Microbiol..

[54] Palza H. (2015). Antimicrobial Polymers with Metal Nanoparticles. Int. J. Mol. Sci..

[55] Al-Tememe M.A.Z., Abdalmoohsin G.R., Mohammadalli T.M., Al Mosawy M.M., Al-Masoudi M.Z. (2019). Molecular Diagnosis of the Fungus Sclerotinia sclerotiorum: A Causal Agent of White Rot Disease in *Solanum melongena* L. and its Control using zinc oxide Nanoparticles. Biopestic. Int..

[56] Alghuthaymi M.A., Kalia A., Bhardwaj K., Bhardwaj P., Abd-Elsalam K.A., Valis M., Kuca K. (2021). Nanohybrid Antifungals for Control of Plant Diseases: Current Status and Future Perspectives. J. Fungi.

[57] Lipovsky A., Nitzan Y., Gedanken A., Lubart R. (2011). Antifungal activity of ZnO nanoparticles—The role of ROS mediated cell injury. Nanotechnology.

[58] Sun Q., Li J., Le T. (2018). Zinc Oxide Nanoparticle as a Novel Class of Antifungal Agents: Current Advances and Future Perspectives. J. Agric. Food Chem..

[59] Faizan M., Faraz A., Yusuf M., Khan S.T., Hayat S. (2018). Zinc Oxide Nanoparticle-mediated Changes in Photosynthetic Efficiency and Antioxidant System of Tomato Plants. Photosynthetica.

[60] Adrees M., Khan Z.S., Hafeez M., Rizwan M., Khalid Hussain K., Asrar M., Alyemeni M.N., Wijaya L., Ali S. (2021). Foliar Ex-posure of Zinc Oxide Nanoparticles Improved the Growth of Wheat (*Triticum aestivum* L.) and Decreased Cadmium Concen-tration in Grains Under Simultaneous Cd and Water Deficient Stress. Ecotox. Environ. Saf..

[61] Rico C.M., Peralta-Videa J.R., Gardea-Torresdey J.L. (2015). Chemistry, Biochemistry of Nanoparticles, and their Role in Antioxidant Defense System in Plants. Nanotechnology and Plant Sciences.

[62] Rossi L., Fedenia L.N., Sharifan H., Ma X., Lombardini L. (2019). Effects of Foliar Application of Zinc Sulfate and Zinc Nanopar-ticles in Coffee (*Coffea arabica* L.) Plants. Plant Physiol. Biochem..

[63] Luksiene Z., Rasiukeviciute N., Zudyte B., Uselis N. (2020). Innovative approach to sunlight activated biofungicides for strawberry crop protection: ZnO nanoparticles. J. Photochem. Photobiol. B Biol..

[64] Petit A.-N., Fontaine F., Vatsa P., Clément C., Vaillant-Gaveau N. (2012). Fungicide impacts on photosynthesis in crop plants. Photosynth. Res..

[65] Larue C., Castillo-Michel H., Sobanska S., Trcera N., Sorieul S., Cécillon L., Ouerdane L., Legros S., Sarret G. (2014). Fate of pristine TiO_2_ nanoparticles and aged paint-containing TiO_2_ nanoparticles in lettuce crop after foliar exposure. J. Hazard. Mater..

[66] Su Y., Ashworth V., Kim C., Adeleye A.S., Rolshausen P., Roper C., White J., Jassby D. (2019). Delivery, uptake, fate, and transport of engineered nanoparticles in plants: A critical review and data analysis. Environ. Sci. Nano.

[67] Lv J., Christie P., Zhang S. (2019). Uptake, Translocation, and Transformation of Metal-based Nanoparticles in Plants: Recent Ad-vances and Methodological Challenges. Environ. Sci. Nano.

[68] Dimkpa C.O., Latta D.E., McLean J.E., Britt D.W., Boyanov M.I., Anderson A.J. (2013). Fate of CuO and ZnO Nano and Micro Par-ticles in the Plant Environment. Environ. Sci. Technol..

