# Supplementary material for: CuZn and ZnO Nanoflowers as Nano-Fungicides against Botrytis cinerea and Sclerotinia sclerotiorum: Phytoprotection, Translocation, and Impact after Foliar Application
Source: Materials (Basel). 2021 Dec 10;14(24):7600. doi: 10.3390/ma14247600 (PMC8708589; doi:10.3390/ma14247600)
Supplement: Supplementary file 1 [file materials-14-07600-s001.zip › materials-1477283-supplementary.pdf]

materials

MDPI

Article

CuZn and ZnO Nanoflowers as Nano-Fungicides against *Botrytis cinerea* and *Sclerotinia sclerotiorum:* Phytoprotection, Translocation, and Impact after Foliar Application

Panagiota Tryfon [1], Nathalie N. Kamou [2], Stefanos Mourdikoudis [3,4], Katerina Karamanoli [5], Urania Menkissoglu-Spiroudi [2,*] and Catherine Dendrinou-Samara [1,*]

[1] Laboratory of Inorganic Chemistry, Department of Chemistry, Aristotle University of Thessaloniki, Thessaloniki 54124, Greece; tryfon.giota@gmail.com

[2] Pesticide Science Laboratory, School of Agriculture, Faculty of Agriculture Forestry and Natural Environment, Aristotle University of Thessaloniki, 54124 Thessaloniki, Greece; nnkamou@gmail.com

[3] Biophysics Group, Department of Physics and Astronomy, University College London, London WC1E 6BT, UK

[4] UCL Healthcare Biomagnetics and Nanomaterials Laboratories, 21 Albemarle Street, London W1S 4BS, UK; s.mourdikoudis@ucl.ac.uk

[5] Laboratory of Agricultural Chemistry, Faculty of Agriculture, Forestry and Natural Environment, Aristotle University of Thessaloniki, 54124 Thessaloniki, Greece; katkar@auth.gr

* Correspondence: rmenkis@agro.auth.gr (UM-S.); samkat@chem.auth.gr (C.D.-S.)

Citation: Tryfon, P.; Kamou, N.N.; Mourdikoudis, S.; Karamanoli, K.; Menkissoglu-Spiroudi, U.; Dendrinou-Samara, C. CuZn and ZnO Nanoflowers as Nano-Fungicides against *Botrytis cinerea* and *Sclerotinia sclerotiorum:* Phytoprotection, Translocation, and Impact after Foliar Application. *Materials* **2021**, *14*, 7600. https://doi.org/10.3390/ma14247600

Academic Editor: Gaurav Sharma

Received: 8 November 2021
Accepted: 7 December 2021
Published: 10 December 2021

Publisher's Note: MDPI stays neutral with regard to jurisdictional claims in published maps and institutional affiliations.

Copyright: © 2021 by the authors. Licensee MDPI, Basel, Switzerland. This article is an open access article distributed under the terms and conditions of the Creative Commons Attribution (CC BY) license (http://creativecommons.org/licenses/by/4.0/).

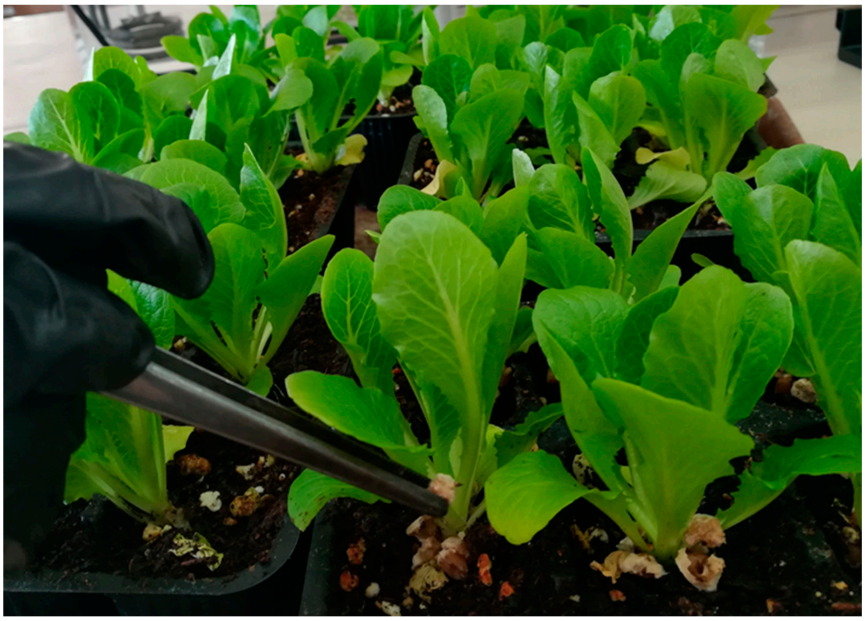

Figure S1. Wheat grains were putted around and next to the wounded stems of lettuce (*Lactuca sativa*) plants.

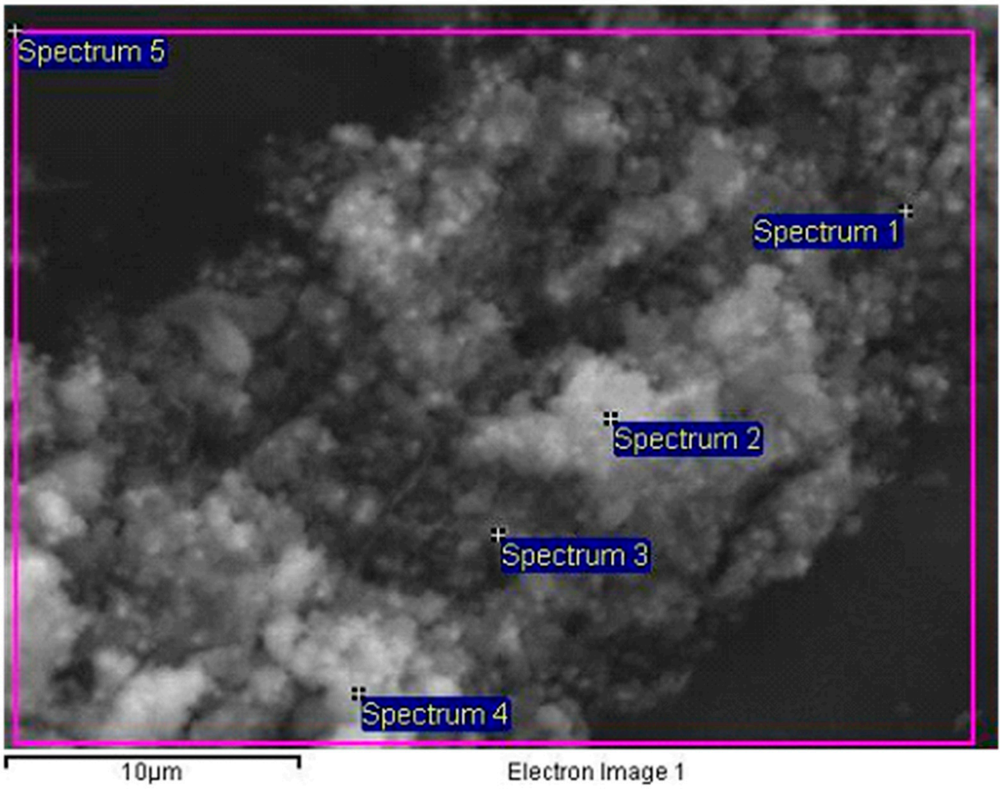

Figure S2. SEM image of the synthesized CuZn@DEG NFs with the five spectrum areas used for the calculation of the elemental composition by EDX.

Table S1. Elemental composition percentage of the CuZn@DEG NFs calculated by EDX for the spectrum areas shown in the SEM image (Figure S2).

Spectrum	Cu	Zn
1	70.42%	29.58%
2	66.73%	33.27%
3	82.48%	17.52%
4	71.75%	28.25%
5	62.21%	37.79%
Mean	70.72%	29.28%
SD	7.55%	7.55
Max.	82.48%	37.79
Min.	62.21%	17.52

CuZn@DEG NPs

			Size (d.nm):	% Intensity	Width (d.nm):
Z-Average (d.nm):	306.4	Peak 1:	190.9	100.0	38.80
PdI:	0.237	Peak 2:	0.000	0.0	0.000
Intercept:	0.904	Peak 3:	0.000	0.0	0.000

Result quality : Good

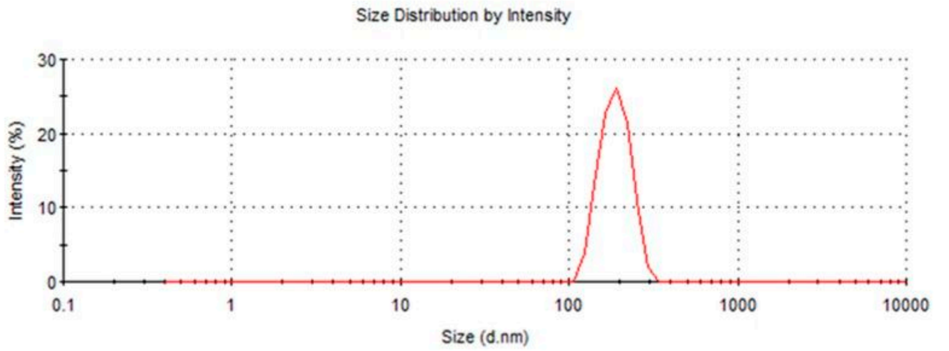

ZnO@PEG NPs

			Size (d.nm):	% Intensity	Width (d.nm):
Z-Average (d.nm):	338.0	Peak 1:	222.7	92.5	66.87
PdI:	0.402	Peak 2:	74.00	7.5	12.72
Intercept:	0.723	Peak 3:	0.000	0.0	0.000

Result quality : Good

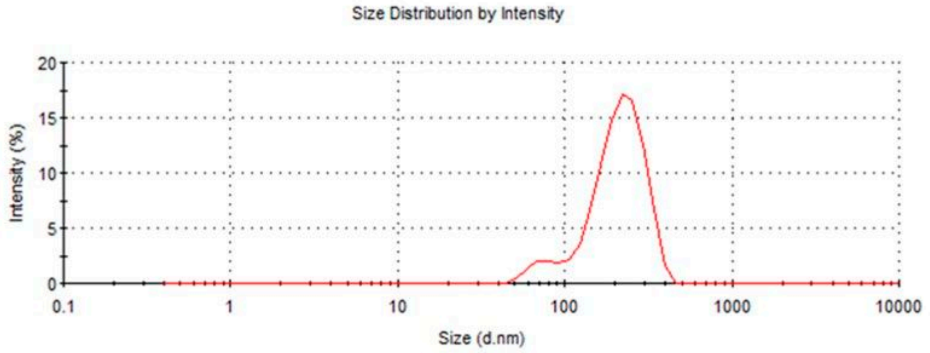

Figure S3. DLS measurements for CuZn@DEG and ZnO@PEG NFs.

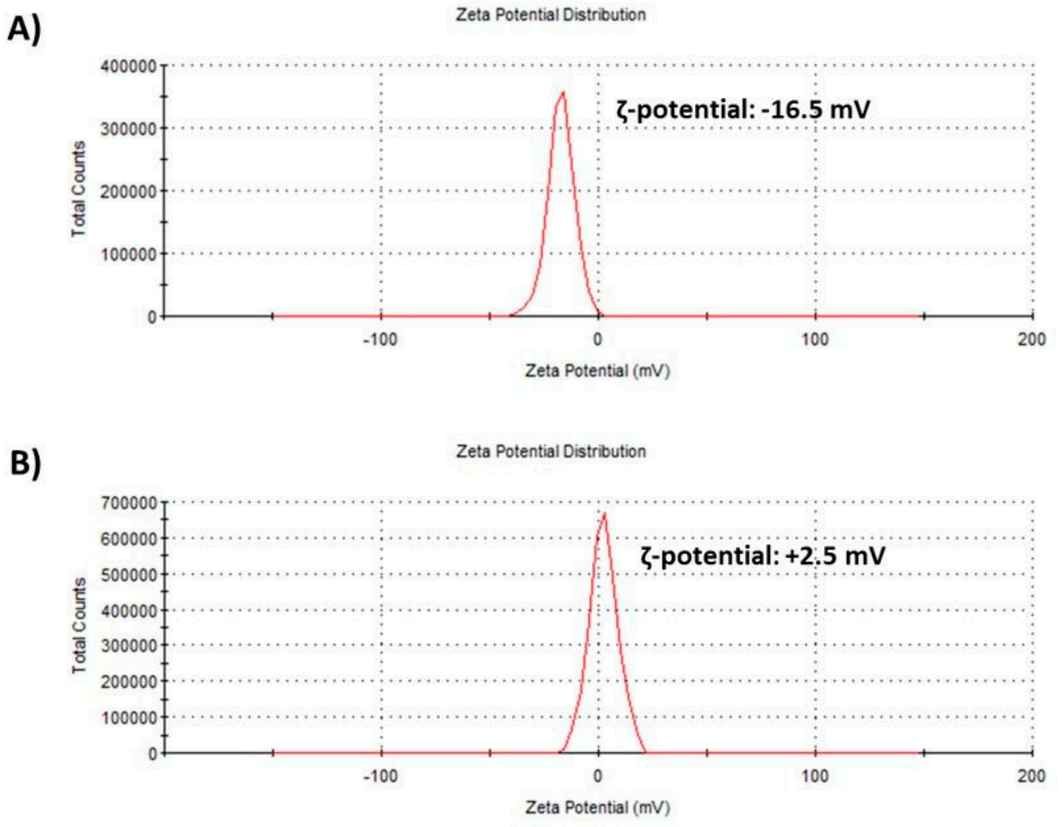

Figure S4. ζ-potential for CuZn@DEG (**A**) and ZnO@PEG (**B**) NFs.

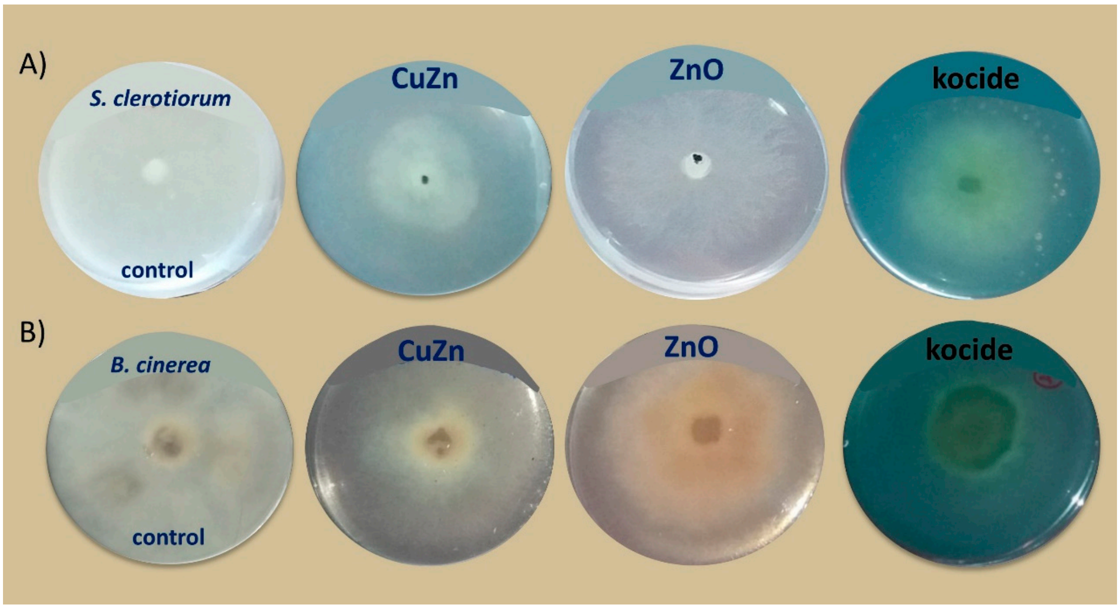

Figure S5. Growth inhibition of *S. sclerotiorum* after 72 h (**A**) and *B. cinerea* after 96 h (**B**) treated with CuZn@DEG NFs, ZnO@PEG NFs and KOCIDE 2000. .
